# Pathology of Experimental *Encephalitozoon cuniculi* Infection in Immunocompetent and Immunosuppressed Mice in Iraq

**DOI:** 10.1155/2014/857036

**Published:** 2014-03-20

**Authors:** Hafidh I. Al-Sadi, Saevan S. Al-Mahmood

**Affiliations:** ^1^Department of Oral and Maxillofacial Surgery, College of Dentistry, University of Mosul, Mosul, Iraq; ^2^Department of Pathology and Poultry Diseases, College of Veterinary Medicine, University of Mosul, Mosul, Iraq

## Abstract

This study was performed to evaluate pathology of experimental *Encephalitozoon cuniculi* (Iraqi isolate) infection in normal and immunosuppressed mice. Pathological changes were not seen in negative control mice while secondary bacterial infections were noted in the lungs, kidneys, and heart of mice given dexamethasone. Typical *E. cuniculi* infection lesions were found in brain, livers, lungs, and kidneys of mice given 10^7^ *E. cuniculi* spores/mouse orally. These lesions were in the form of nonsuppurative meningoencephalitis with vasculitis in brain, interstitial inflammation with infiltration of both lymphocytes and plasma cells in lung tissue, and nonsuppurative interstitial (focal and diffuse) nephritis, presence of vacuole containing mature and immature spores in enterocytes within the tips of villi, and lymphoiod hyperplasia of the white pulp and vasculitis of the intratrabecular vessels. Mice that were given 10^7^ *E. cuniculi* spores/mouse orally showed lesions similar to those observed in the previous group (vasculitis and granulomas) but the lesions were more severe and widespread. In conclusion, this is the first report of experimental *E. cuniculi* infection induced by *E. cuniculi* isolated from a naturally infected rabbit in Iraq and that infection became more severe and widespread upon the administration of dexaethasone.

## 1. Introduction

Microsporidia are obligate intracellular microorganisms that infect a wide range of invertebrate and vertebrate species [1]. Traditionally, they were considered protozoa but recently have been reclassified phylogenetically as fungi [2]. These microorganisms have been reported as causing economic losses in honeybee, fish, mink, and other fur-bearing animals [3].

The phylum Microsporidia is subdivided into a variety of families and genera, which includes the genus* Encephalitozoon* which in turn includes the important species* Encephalitozoon cuniculi*,* E. hellem*,* E. intestinalis*, and* E. lacerate* [4]. Microsporidian spores have been reported in foods and water sources [3]. The natural route of entry of microsporidia into the host was found to be through ingestion or inhalation of infective spores or via wounds and transplacentally [3].

*E. cuniculi* has been considered the most common and most important pathogen in animals; most of the knowledge available now on microsporidia is based on* E. cuniculi* [5]. Additionally,* E. cuniculi* was also the first mammalian microsporidium that was isolated and cultured in vitro [6].

In immunocompetent host, microsporidial infection is characterized by a short acute diarrheal phase followed by asymptomatic infection, while chronic malabsorptive diarrhea and systemic disease can develop in immunocompromised individuals [7].

Although microsporidia have been known as pathogenic agents in a wide range of wild, laboratory, and domestic animals for several decades, further studies are required to completely understand the epidemiology and pathobiology of microsporidiosis; also these parasites have not been isolated or identified before in Iraq; furthermore, their pathology in different species was not studied. The present study was designed to determine the pathology of experimental* E. cuniculi* (using* E. cuniculi* isolated from rabbit infected in Iraq) in immunocompetent and immunosuppressed mice.

## 2. Materials and Methods

### 2.1. Ethics Statement

All of the experimental procedures were conducted in accordance with the regulations of the College of Veterinary Medicine, University of Mosul, concerning protection of animals against cruelty.

### 2.2. Experimental Animals

A total of 240 inbred Balb/c, 35- to 42-day-old male mice were housed in the Animal House of the College of Veterinary Medicine, University of Mosul, given food and water ad libitum, and maintained on a 12-hour light/dark cycle. Mice were allowed to acclimate for one week prior to their use in the experiments. To prevent reinfection, microisolator cages, feed, water, and bedding were sterilized and changed daily. All animals were tested daily (fecal examination) for the presence of microsporidia. Only mice without microsporidial infection were used. Additionally, mice were treated orally with albendazol (25 mg/kg b.w.) and five days later with 0.5 mg/kg b.w. of fumagillin to get rid of any possible microsporidial or other parasitic infections [8].

### 2.3. Preparation of Inoculum

Spores of microsporidia (*Encephalitozoon cuniculi*) were obtained from fecal samples of a naturally infected rabbit isolated by Al-Sadi and Al-Mahmood [9]. Infected spores of* E. cuniculi *were prepared as described by [10].

### 2.4. Experimental Design

Mice (*n* = 240) were divided into 4 equal groups randomly: first group (control group, nontreated), second group (microsporidia group) which was given 10^7^ spores of* E. cuniculi*/mouse orally, third group (*E. cuniculi* and dexamethasone) which was given 10^7^ *E. cuniculi* spores/mouse orally and given dexamethasone 125 *μ*g/mouse intraperitoneally during the first day of the experimentation and repeated once a week for the rest of the experimental period, and fourth group (control group, dexamethasone treated) which was given dexamethasone 125 *μ*g/mouse intraperitoneally during the first day of the experimentation and repeated once a week for the rest of the experimental period [11]. Fecal samples were collected from mice at 3, 7, 10, 15, 30, and 60 postinoculation days (PID). Smears were prepared from the samples and the slides were stained with Giemsa, quick-hot Gram-chromotrope, Weber-green modified trichrome, Ryan-blue modified trichrome, and Calcofluor-white stains [12–15]. Slides were examined under a light microscope to determine the presence of* E. cuniculi *spores and to determine the mean shedding of microsporidia.

### 2.5. Histopathological Examination

Ten mice from each group were euthanized at each of the 3rd, 7th, 10th, 15th, 30th, and 60th postinoculation days using chloroform [16]. Following gross pathological examination, tissue specimens were collected from the brain, kidneys, liver, lungs, intestines, pancreas, heart, and spleen; these tissue specimens were fixed as described by [15]. Following fixation these tissues were processed and stained by routine stain as described by [10, 12, 15].

## 3. Results

**Figure 1:**
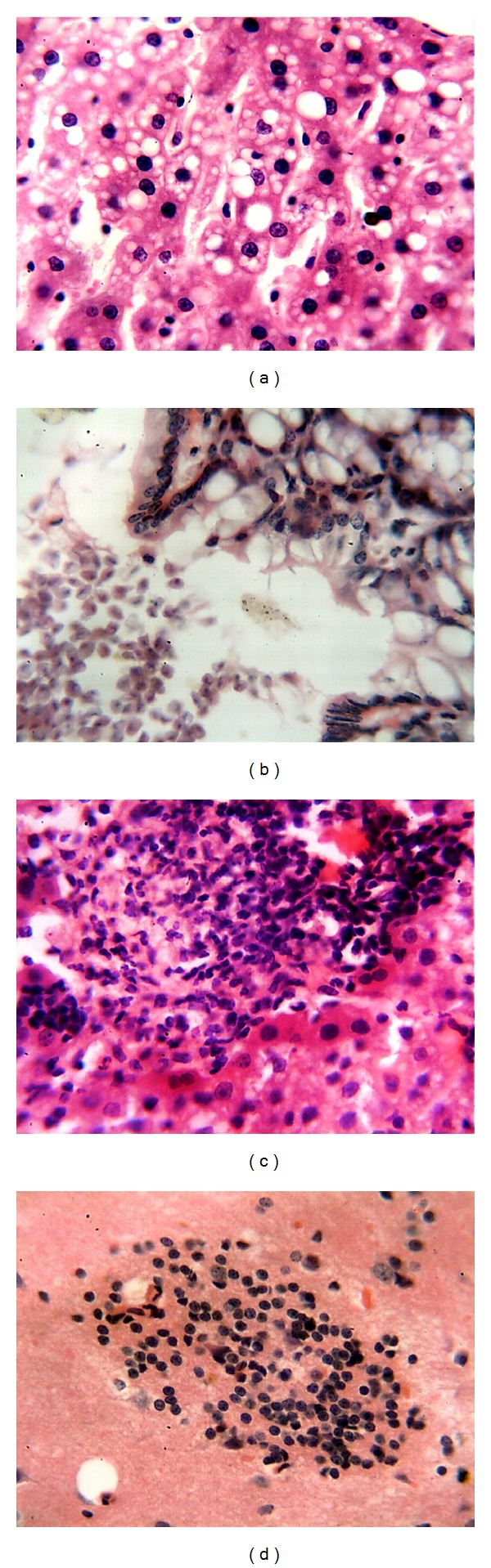
(a) Vacuolar degeneration of hepatocytes; (b)* E. cuniculi* spores in intestinal lumen; (c) hepatic granuloma; (d) glial nodule in the cerebral cortex. All HE; 400x.

**Figure 2:**
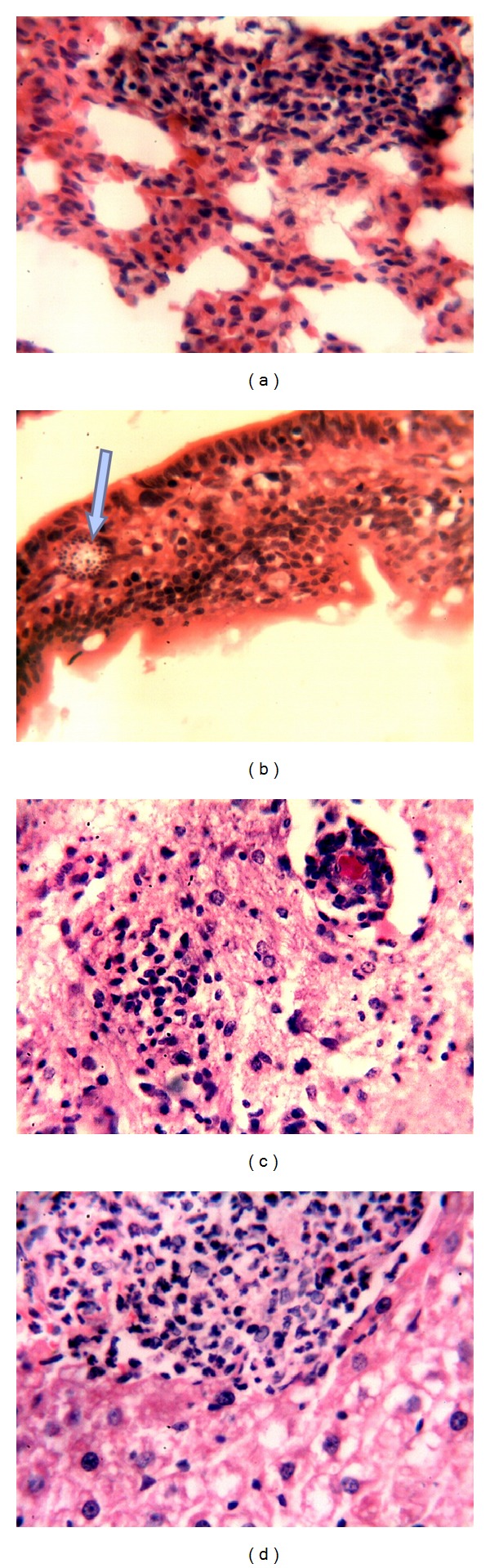
(a) Granulomatous lesion in the lung; (b) parasitophorous vacuole containing spores of* E. cuniculi* in enterocyte (arrow); (c) cerebral granuloma; (d) hepatic granuloma. All HE; 400x.

There were no significant clinical signs or gross lesions in immunocompetent mice inoculated with* E. cuniculi*, at the third PID. Microscopically, renal tissues exhibited focal infiltration of mononuclear cells in the interstitium. Fatty change of hepatocytes and mild vasculitis were found within the portal areas (Figure 1(a)). Pulmonary tissue showed heavy infiltration of mononuclear cells. In the lumen of intestines, large numbers of spores were detected (Figure 1(b)). Similar changes were found at the 7th PID. However, at the 10th PID, focal granulomatous inflammation and vasculitis were seen in the hepatic parenchyma and lymphocytic cholangitis was found in the portal areas (Figure 1(c)). The lungs exhibited vasculitis in the interstitium and lymphoid hyperplasia of the peribronchial lymph nodes. Other lesions included lymphocytic enteritis and granulomatous splenitis. At the 15th PID, the brain showed vasculitis in the meninges, gliosis in cerebral cortex, and granulomas in the cerebrum (Figure 1(d)). The lungs showed vasculitis and granulomatous pneumonia (Figure 2(a)). In the intestines, the parasites were seen in parasitophorous vacuoles in enterocytes within the villi (Figure 2(b)). At the 30th PID, grossly, there were congestions of meninges, brain, liver, and spleen. The lungs, stomach, and intestines were pale with presence of hemorrhagic area in the lungs. Microscopically, the nervous tissue showed vasculitis and granulomas in the cerebrum and vasculitis in the cerebellum (Figure 2(c)). At the 60th PID, the mice were anorexic and depressed. Grossly, there were congestions of meninges, brain, and liver and paleness of intestines and lungs. The latter also exhibited scattered nodular lesions. Histologically, granulomas were seen in the liver as well as thickening of blood vessels and bile ducts in portal areas (Figure 2(d)).

**Figure 3:**
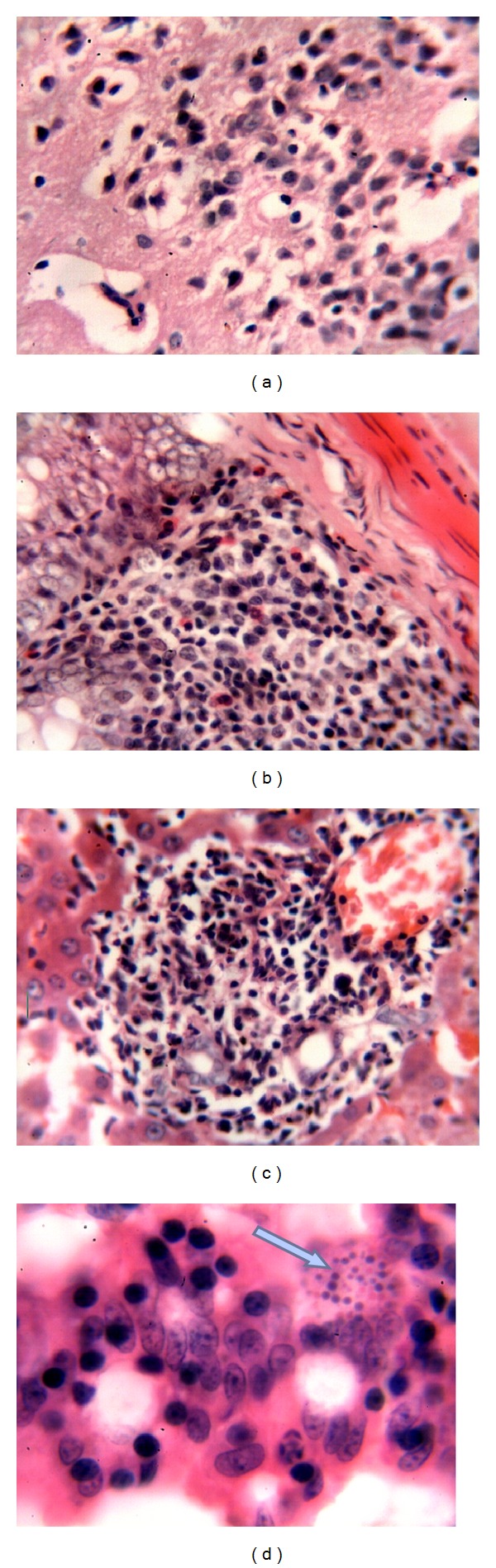
(a) Focal infiltration of inflammatory mononuclear cells in the cerebrum; (b) lymphoid hyperplasia in intestinal submucosa; (c) hepatic granuloma; (d)* E. cuniculi* spores in enterocytes (arrow). All HE; 400x.

**Figure 4:**
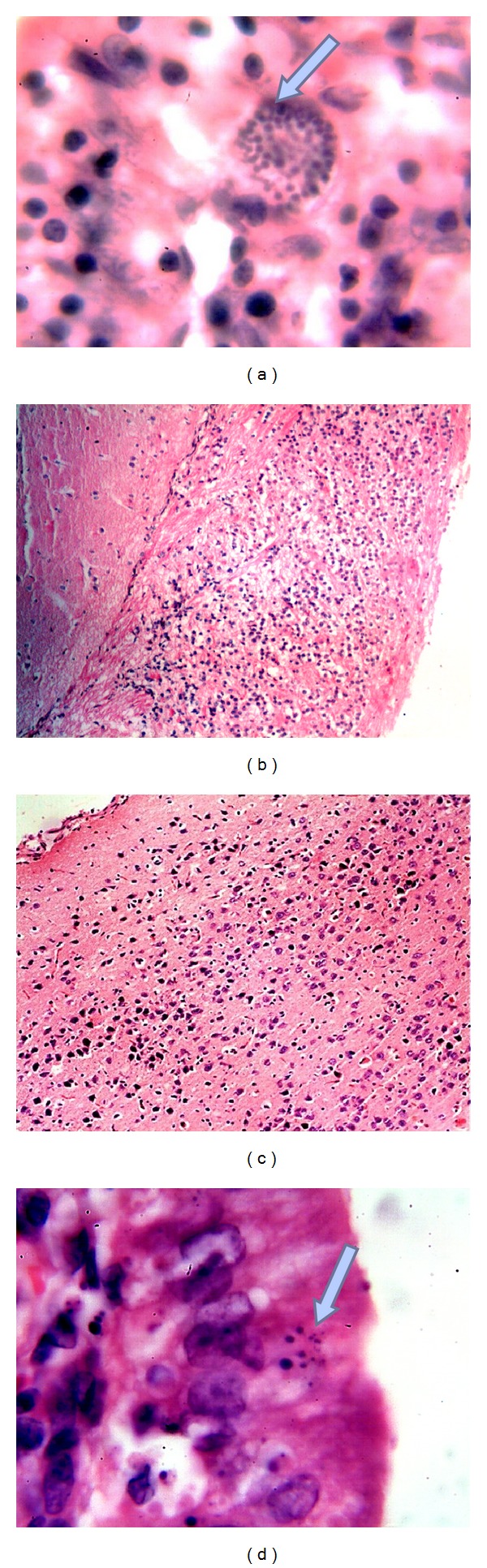
(a)* E. cuniculi* spores in parasitophorous vacuole in enterocytes (arrow) (1000x); (b) heavy infiltration of inflammatory mononuclear cells in the meninges and cerebral cortex (100x); (c) diffuse infiltration of inflammatory mononuclear cells in the cerebral cortex (100x); (d)* E. cuniculi* spores in enterocytes (arrow) (1000x). All HE.

In immunosuppressed mice, at the 7th PID, microscopically, there was infiltration of mononuclear cells in cerebral cortex and cerebellum (Figure 3(a)). Hyperplasia of the lymphoid follicles was seen in the submucosa of the intestines (Figure 3(b)). At the 10th PID, anorexia, sneezing, coughing, and staggering of mice were seen. Vasculitis, cholangitis, and granulomas were seen in the liver (Figure 3(c)). The intestines showed lymphocytic enteritis and the presence of the parasites in parasitophorous vacuoles in enterocytes (Figure 3(d)). At the 15th PID, the parasite was seen in parasitophorous vacuoles in enterocytes (Figure 4(a)). At the 30th PID, microscopically, vasculitis and heavy infiltration of mononuclear cells were found in meninges, cerebrum, and cerebellum (Figure 4(b)), and vasculitis and focal interstitial nephritis were noted. At the 60th PID, microscopically, diffuse mononuclear cell infiltrations were seen in brain and meninges (Figure 4(c)). In the intestines, there were lymphocytic enteritis, thickening of the serosa due to proliferation of smooth muscle, and mesothelial cells and presence of the parasites in enterocytes (Figure 4(d)).

## 4. Discussion

In the present study,* E. cuniculi* spores of rabbit origin isolated in Iraq were used to induce the infection in mice, and a special emphasis was made on pathology of the infection in immunocompetent and immunosuppressed mice. Previously, there are no studies on the presence or occurrence and pathology of microsporidial species in Iraq, in both human and animals, which makes it impossible to compare this study with others in Iraq and also makes the current study the first one that identified and diagnosed presence of these microorganisms in Iraq.

Two basic patterns of encephalitozoonosis are known to occur, an acute and clinically detectable disease, frequently resulting in death. This form has been induced in neonates as well as immunocompromised animals [17, 18]. The second form is recognized as a chronic and silent infection, which is mostly seen in healthy animals [17–19].

In second group, histological lesions seen at seven days after infection were in the form of mononuclear inflammatory foci in the brain, liver, lungs, kidneys, and intestines. After that and up to the sixty days after infection, the lesions included vasculitis, gliosis and granulomas of the cerebral cortex, focal interstitial nephritis, vasculitis cholangitis and granulomas in the liver, granulomas in the lungs, enteritis, and depletion of lymphocytes from the germinative centers of the spleen. These lesions have been attributed to subclinical* E. cuniculi* infection; they are similar to what have been found by [20, 21]. Infection with the* E. cuniculi* has been shown to originate in either the intestinal or respiratory epithelia; dissemination of* E. cuniculi* can cause nephritis as well as infections of the sinuses, urinary bladder, and skin;* E. cuniculi* has been also shown to cause keratoconjunctivitis and is capable of infecting the heart, brain, kidneys, and even the tongue; this is due to its ability to disseminate to other parts of the body for the ability of these species to infect macrophages and be transported to other organs and tissues.

Immunosuppressed mice infected with* E. cuniculi* showed similar clinical signs and gross lesions to those exhibited by immunocompetent mice. However, at the ten days after infection necrotic areas were seen in the lungs, and at the sixty days after infection nodular lesions raised over the surface of the lung and hearts were found. Histological lesions included vasculitis and granulomas in meninges and brain, vasculitis and hyperplasia of the peribronchial lymphoid follicles, purulent bronchopneumonia and pleuritis, focal interstitial nephritis, vasculitis, cholangitis and granulomas in the liver, enteritis, and presence of numerous macrophages and cellular debris in the spleen. These changes were similar to those described in experimental infection in athymic nude or severely combined immunodeficient mice [22–24]. Many studies concluded that resistance to infection was mediated by one or more cytokines but not antibody responses; many studies reported that the infection with* E. cuniculi* is dependent on the secretion of IL-12 and INF-*γ* and then the role of T_**1**_ helper lymphocytes in clearing the pathogens from these organs, this is manifested from infection of* E. cuniculi* to INF-*γ* null mice and IL-12 deficient mice could not eliminate the infection without presence of T_**1**_ helper lymphocytes [25], so the following sentence is clearly right: resistance or susceptibility to* E. cuniculi* infection is interdependent upon interactions of T_**1**_ helper lymphocytes with the innate arm of immunity.

It has been shown previously that immunosuppression in mice with 100 mg/kg of cyclophosphamide, twice a week by intraperitoneal route, produced an acute form of encephalitozoonosis, recognized as a disseminated and lethal infection [26]. In another study, immunosuppression was induced using intraperitoneal injection of 50 mg/kg of cyclophosphamide twice a week and the infection was chronic [17]. In the present study, the dose schedule of dexamethasone 125 *μ*g/mouse intraperitoneally once a week for 9 weeks leads to the occurrence of chronic infection, and this effect of immunosuppression of dexamethasone is studied widely by other researchers in which they recommended the dose of 125 *μ*g/mouse intraperitoneally once a week to overcome the immune system of mice [11], so it does not need any evaluation of the immunosuppression achieved with the use of dexamethasone [11, 27].

The present study provided evidence that mice and rabbit isolates of* E. cuniculi* are identical and that any difference in the nature and distribution of lesions in the two species may be due to dissimilarities in the hosts. These findings were in accordance with those reported by others [28].

## 5. Conclusions

In this study, the microsporidial species generally and* Encephalitozoon cuniculi* especially are present in Iraq. This study is considered the first one to report its presence and pathology in Iraq. Also the pathological lesions in both immunocompetent and immunosuppressed mice were typical of* Encephalitozoon cuniculi* infection; however, the lesions were more severe, extensive, and widespread in immunosuppressed mice.
